# Genetic and context‐specific effects on individual inhibitory control performance in the guppy (*Poecilia reticulata*)

**DOI:** 10.1111/jeb.14241

**Published:** 2023-11-02

**Authors:** Pamela M. Prentice, Alex Thornton, Niclas Kolm, Alastair J. Wilson

**Affiliations:** ^1^ Centre for Ecology and Conservation University of Exeter Penryn UK; ^2^ SRUC, Easter Bush, Roslin Institute Building Midlothian UK; ^3^ Department of Zoology Stockholm University Stockholm Sweden

**Keywords:** behaviour, cognition, guppy, heritability, Quantitative genetics

## Abstract

Among‐individual variation in cognitive traits, widely assumed to have evolved under adaptive processes, is increasingly being demonstrated across animal taxa. As variation among individuals is required for natural selection, characterizing individual differences and their heritability is important to understand how cognitive traits evolve. Here, we use a quantitative genetic study of wild‐type guppies repeatedly exposed to a ‘detour task’ to test for genetic variance in the cognitive trait of inhibitory control. We also test for genotype‐by‐environment interactions (GxE) by testing related fish under alternative experimental treatments (transparent vs. semi‐transparent barrier in the detour‐task). We find among‐individual variation in detour task performance, consistent with differences in inhibitory control. However, analysis of GxE reveals that heritable factors only contribute to performance variation in one treatment. This suggests that the adaptive evolutionary potential of inhibitory control (and/or other latent variables contributing to task performance) may be highly sensitive to environmental conditions. The presence of GxE also implies that the plastic response of detour task performance to treatment environment is genetically variable. Our results are consistent with a scenario where variation in individual inhibitory control stems from complex interactions between heritable and plastic components.

## Graphical abstract

We use a quantitative genetic study to examine variation in cognitive performance in wild‐type guppies. We show among‐individual variation in detour task performance, consistent with differences in inhibitory control as well as plasticity to visual information context. Heritable variation was also detected but GxE means this is limited to the low information treatment. Our results suggest the adaptive evolutionary potential of inhibitory control may be highly sensitive to environmental context.

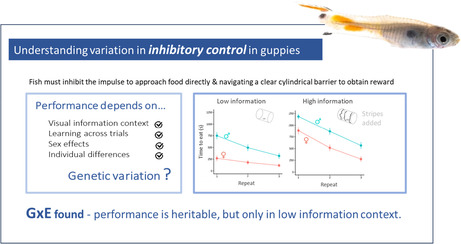

## 1 INTRODUCTION

Cognitive traits are the neural mechanisms by which animals acquire, process, store, and use information from their environment (Healy & Rowe, 2010; Shettleworth, 2010). While the evolution of cognition has long been studied using comparative approaches [for a review see (Healy, 2019)], more recent advancements have aimed to characterize variation in cognitive performance among individuals within populations (Boogert et al., 2018). Variation among‐individuals is required for natural selection (Roff, 2002), and a heritable component to this variation is necessary for an evolutionary response (Croston et al., 2015; Thornton & Wilson, 2015). Here, we test both these conditions using a quantitative genetic study of wild‐type guppies repeatedly exposed to a ‘detour task’ in which differences in performance are expected to arise from cognitive processes including, but not limited to, inhibitory control (Kabadayi et al., 2018).

Recent studies show variation in cognitive performance among conspecifics within populations [for reviews see (Boogert et al., 2018; Cauchoix et al., 2018)]. This pattern is seen across taxa, from insects [e.g., (Li et al., 2017; Pull et al., 2022)], fish [e.g., (Buechel et al., 2018; Prentice, Mnatzaganian, et al., 2020)], to mammals [e.g., (Mazza et al., 2018; Nawroth et al., 2017) and birds [e.g., (Branch et al., 2020; Guillette et al., 2015)], and across different aspects, or ‘domains’, of cognition [e.g., spatial memory (Sonnenberg et al., 2019); spatial leaning (White et al., 2017), inhibitory control (van Horik et al., 2019)]. The fitness consequences of such variation remain unclear and are clearly difficult to infer from cognitive studies under controlled laboratory conditions. However, at least some field‐based studies (Cole et al., 2012; Raine & Chittka, 2008) suggest cognitive variation has fitness consequences and is subject to positive directional natural selection. For example, reproductive success in female Australian magpies (*Cracticus tibicen dorsalis*) is positively associated with high cognitive performance across multiple domains (Ashton et al., 2018), while male New Zealand robins (*Petroica longipes*) performing well in a spatial learning task produced more fledglings with higher survival (Shaw et al., 2019). Similarly, in mountain chickadees (*Poecile gambeli*), spatial learning and memory are positively associated with overwinter survival (Sonnenberg et al., 2019). While such studies provide support for positive selection acting on cognitive traits currently, genomic studies have suggested strong selection in the past. For instance, the expectation of reduced molecular variation in the vicinity of a beneficial mutation spreading rapidly through a population (Smith & Haigh, 1974), has led to inference of positive selection on genes associated with cognition and learning in great tits (*Parus major)* (Laine et al., 2016), and face recognition in paper wasps (*Polistes fuscatus*) (Miller et al., 2020).

Among‐individual variation in cognitive performance is therefore widespread and likely to have fitness consequences (Ashton et al., 2018; Corral‐López et al., 2017; Shaw et al., 2019). These are two of the three conditions required for adaptive phenotypic evolution (Wilson et al., 2010), the third being the presence of heritable genetic factors. However, relatively few studies have quantified the genetic contribution to among‐individual variance in animal cognition traits (Branch et al., 2020; Hopkins et al., 2014; Langley et al., 2020; Sorato et al., 2018; Vardi et al., 2020). Results from empirical studies to date also yield somewhat mixed findings. For example, genetic variation contributes to differences in reversal learning in red junglefowl (*Gallus gallus*), but not towards differences in performance when individuals are trained to discriminate between rewarded and unrewarded cues (Sorato et al., 2018). In pheasants (*Phasianus colchicus*) (Langley et al., 2020), heritability estimates are low for some tasks (e.g., spatial learning, *h* ^2^ = 0.09), but moderate for others (e.g., discrimination learning, *h* ^2^ = 0.17; inhibitory control, *h* ^2^ = 0.23). Artificial selection experiments on brain size and structure have also yielded responses accompanied by evolved changes in cognitive performance (Buechel et al., 2018; Fong et al., 2021; Triki et al., 2022). While these examples show that genes can be important for some cognitive traits, other studies have found less support. For example, problem‐solving within a foraging task was not heritable in wild great tits (Quinn et al., 2016), and nor was spatial learning in delicate skinks (*Lampropholis delicate*) tested using a Y maze paradigm (Fong et al., 2021). More studies are needed before we can determine if there are systematic differences in cognitive trait heritabilities among domains, taxa or types of study (e.g., controlled laboratory studies versus in situ estimates in wild animals).

Additionally, since cognition is defined in relation to acquiring, processing, storing and using information *from the environment*, genotype‐by‐environment interactions (GxE) may be common. GxE means the genotype–phenotype map is environmentally sensitive. This means quantitative genetic variance depends on conditions (Mackay, 1981; Nussey et al., 2007; Roff, 2012), such that, for example, genetic variance for performance in a cognitive assay will vary with the information context (e.g., amount of visual information availability; (Pike et al., 2018). Alternatively, and equivalently, GxE can be understood as genetic variance in plasticity (see 46 for a didactic explanation of this) such that, in the above example, within‐individual change in performance across different information contexts can be viewed as a heritable trait. Although explicit examination of GxE is rare in animal cognition (see (Hunt et al., 2019)), there is compelling evidence from experimental studies on other labile behaviours. For example, calling effort by male crickets depends on GxE across a range of environmental factors (e.g., temperature, diet, social context (Callander et al., 2013; Hedrick et al., 2002; Kasumovic et al., 2012; Rapkin et al., 2017)). Similarly, quantitative genetic studies in the emergent field of animal personality have shown that genetic variation in plasticity (i.e., GxE) can contribute to among‐individual variation in behaviour (Edwards et al., 2017; Rudin et al., 2019; Wey et al., 2019).

In this study, we test for and characterize additive genetic variance and GxE for inhibitory control, which is the ability to inhibit pre‐potent responses (e.g., attempting to move straight towards a reward) in favour of more effective or appropriate behaviours (Kabadayi et al., 2018), in the guppy (*Poecilia reticulata*). This freshwater poecilid is widely used as a model for behavioural genetic (Prentice, Houslay, et al., 2020) and cognitive studies (Fong et al., 2019; Kotrschal et al., 2015; Lucon‐Xiccato & Bisazza, 2016). Previous work has examined variation in learning colour associations (Buechel et al., 2018; Trompf & Brown, 2014), reversal learning (Buechel et al., 2018), numerical discriminations (Kotrschal et al., 2013; Lucon‐Xiccato & Bisazza, 2017a) and spatial association (Kotrschal et al., 2015; Lucon‐Xiccato & Bisazza, 2017a; Prentice, Mnatzaganian, et al., 2020). Inhibitory control has also been reported in this species (Lucon‐Xiccato & Bisazza, 2016), and can vary among‐individuals (Macario et al., 2021; Triki et al., 2022) though the extent to which genetic factors underpin this is currently unknown. Nor is it known what fitness consequences, if any, variation in inhibitory control has for wild guppies. Animals can benefit from inhibiting behaviours under some conditions, for example, reducing foraging or parental care when competing conspecific or predator densities are high (Beran, 2015; Fontaine & Martin, 2006; Soltis et al., 2001). However, estimates of natural selection are lacking (Ashton et al., 2018). In humans, we do know that inhibitory control positively predicts other cognitive abilities (Diamond, 2013) as well as academic outcomes in children (Duckworth et al., 2012). Conversely, it is negatively correlated with propensity for antisocial behaviour and drug abuse (Feil et al., 2010; White et al., 1994).

To characterize genetic variation, we couple pedigree‐based quantitative genetic modelling with a ‘detour task’ testing paradigm (Lucon‐Xiccato et al., 2017). Fish were first trained to feed from a green plastic disc placed in a consistent location. They were then repeatedly assayed in a detour task, in which the feeding disc was placed within a transparent cylinder. This required individuals to move away from the visible reward and detour around the transparent obstacle in order to feed. To test for GxE, we subjected half of the fish to the standard detour task using a fully transparent cylinder, while the other half experienced a cylinder marked with lines to provide additional visual information. Note that our goal is to test for a treatment effect on genetic variance. We have no clear directional predictions for effects on mean performance. Although ‘semi‐transparent’ barriers can improve average performance in detour tasks (Juszczak & Miller, 2016; Noland, 2008; Santos et al., 1999; Zucca et al., 2005), exceptions have also been reported (Zucca et al., 2005). Consequently, we consider it possible that markings could prove mildly aversive, inducing neophobia that increases average time to obtain the food reward. Finally, while detour tasks are primarily used to assay inhibitory control (Kabadayi et al., 2018), variation in performance may also arise to some degree from other executive cognitive functions (e.g., working memory, route planning, object permanence) and associative learning of the affordances of the task (van Horik et al., 2018; van Horik et al., 2019). Moreover, performance variation may also arise from differences in motivation, personality or experience (van Horik et al., 2018). We therefore seek—as far as possible—to validate the involvement of cognitive traits by jointly analysing detour task performance with data on time to feed in the training stage of the experiment. This allows us to ask whether among‐individual and/or genetic differences in time to feed in the absence of the cylinder (e.g., due to variation in motivation, personality or associative learning), are sufficient to explain later variation in detour task performance.

## 2 METHODS

### 2.1 Ethics

This work was conducted under the auspices of the UK Animals (Scientific Procedures) Act (1986) with approval of the University of Exeter research ethics committee, under licence from the Home Office (UK) (Licence Number PPL30/3256). Experimental procedures and behavioural assays were developed in accordance with the principles of the three Rs and ASAB guidelines (Buchanan et al., 2020) for use of animals. All periods of handling and emersion were kept to a minimum and only fish deemed healthy and exhibiting normal behaviour were used in trials.

### 2.2 Fish husbandry and breeding

Fish were bred from a captive population of *P. reticulata* housed at the fish laboratory at the University of Exeter's Penryn campus and descended from wild individuals caught in 2017 in the lower Aripo River, Trinidad. The population has been maintained at a population size of several thousand, with no deliberate selection or inbreeding. All fish were fed to satiation twice daily (0800–1000 h and again at 1600–1800 h) using commercial flake food and live *Artemia nauplii*. Water temperature was maintained at 23–24°C in well‐aerated, closed system tank stacks with a 25% water change each week and weekly testing for ammonia, nitrates and nitrites. Lighting was kept at a 12:12 light/dark cycle.

Quantitative genetic analyses require pedigree or relatedness information. Here, we collected behavioural data on an offspring generation of 374 guppies (all tested as adults), produced from six small breeding groups over a period of 4 months. Breeding groups comprised adult fish sampled randomly from stock tanks and housed in 15 L tanks (18.5 cm × 37 cm × 22 cm). Breeding groups had a median size of 9 fish, with an approximate 2:1 female to male ratio. There was some uncontrolled variation around this, as mortalities occurred during the breeding period. These were replaced where possible, but availability was limited for females. This is because we elected to use only females that had been isolated from males for ≥3 months, reducing the possibility that they were carrying viable sperm from previous matings. Females exhibit sperm storage, but there is a strong paternity bias towards freshly inseminated sperm (Gasparini et al., 2018). Thus, use of isolated females reduced the number of (potential) sires for any offspring individual, and thus the complexity of sibship reconstruction from molecular data (described below). In total, 54 adults from the parental generation entered breeding groups. No behavioural data were collected on the parental generation.

Offspring produced in each breeding tank were removed on detection and transferred to separate 2.8 L brood tanks (10 cm × 28 cm × 15 cm). Relationships within broods remain uncertain since offspring detected within breeding tanks could have been born to multiple females over a short time period, and maternal sibships may have mixed paternity. Offspring were raised in these brood tanks to sexual maturity and behaviourally phenotyped over a single extended period once the total offspring generation reached sufficient sample size. Our decision to do this, rather than test groups at a single standardized age, was largely imposed by effective closure of the fish facility and working restrictions imposed during the COVID‐19 pandemic. As a consequence, the age of offspring tested ranged from 4 to 15 months. Although a recent analysis of cognitive ageing in the guppy suggests this is unlikely to affect the results (Boussard et al., 2021), we control for age effects statistically by including brood tank in all models (see below). This accounts for differences in average age (and any other non‐genetic effects) between groups. We acknowledge that, where >1 maternal sibship is present within a brood, there may be some age variation we cannot control for.

### 2.3 Cognitive testing

**FIGURE 1:**
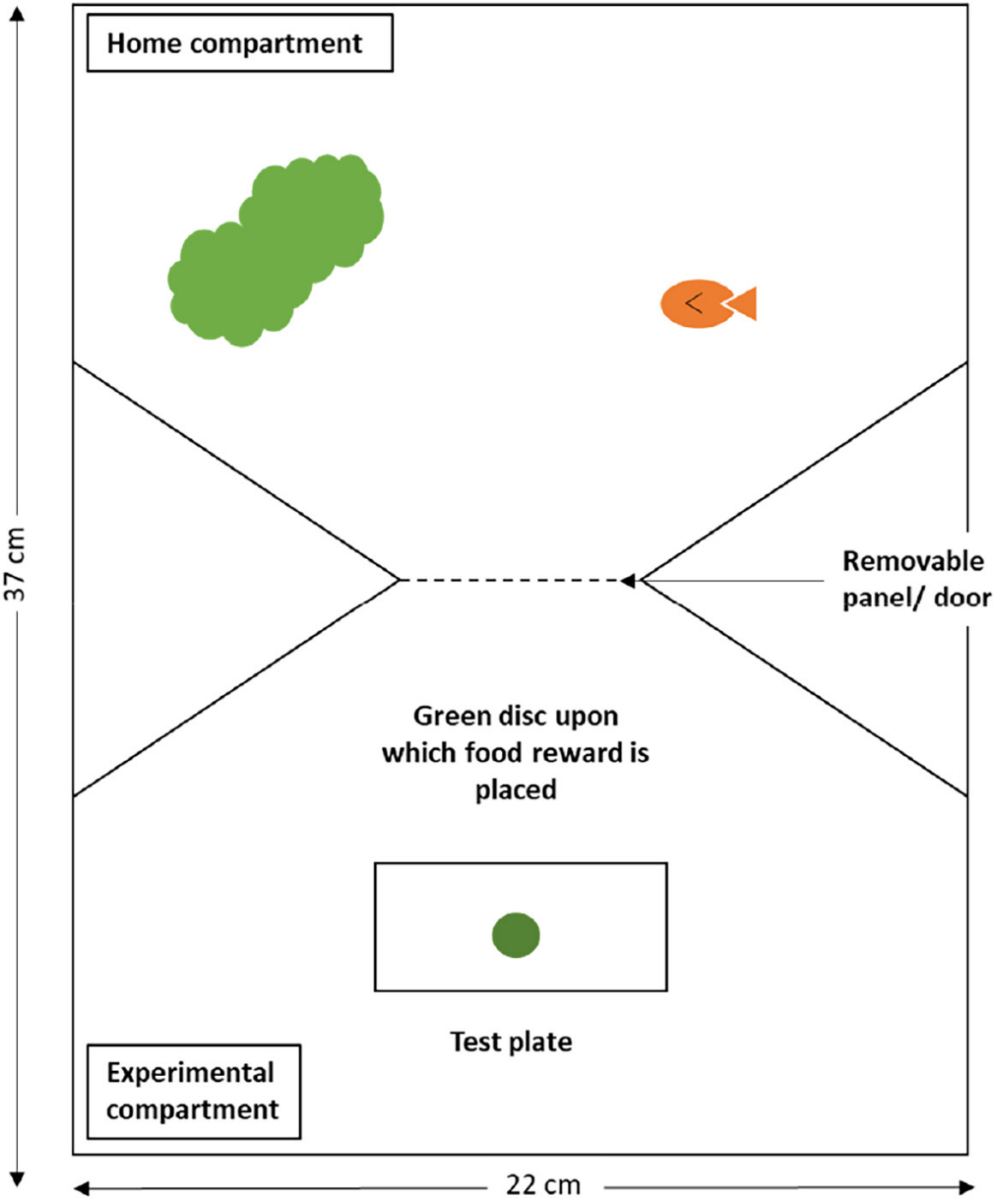
Aerial view of the tank set up used for association training trials and detour task trials.

Individual detour task performance was assessed in a repeated measures design. Guppies were individually transferred into 15 L experimental tanks (18.5 cm × 37 cm × 22 cm) and housed alone for the duration of the behavioural testing. The set‐up of these experimental tanks closely followed that used by (Lucon‐Xiccato & Bisazza, 2014) and (Triki et al., 2022), each being divided into two compartments (using white plastic) separated by a guillotine door (Figure 1). The rear ‘home’ compartment (20 × 18.5 cm) allowed visual access to fish in neighbouring tanks while the front ‘test’ compartment (17 × 18.5 cm) was screened using white plastic to preclude any social learning by observation of neighbours. Individuals were allowed to acclimate for 48 h prior to training and testing. During acclimation, they were fed once a day (with one‐third pipette of live artemia for males, and one pipette for females) and the guillotine door was left open allowing free use of both compartments. Experimental tanks were contained within two ‘stacks’, each comprising 24 tanks (eight tanks per row, three rows high) on a shared recirculating water supply. Thus, data could be collected in a block of up to 48 fish. The testing protocol took 8 days per fish, so in practice this was done in 11 blocks over a total period spanning 13 weeks.

For each individual in each block, behavioural data collection comprised two stages. First, naïve guppies were given the opportunity to learn to associate the appearance of a green disc placed on the floor of the test compartment with a food reward. Individual guppies were given nine feeding training ‘trials’ (three per day for three successive days). To ensure fish had sufficient opportunity to learn the food location, they did not proceed to the detour task if they did not locate food in 5/9 of the training trials. Note, we wanted to assess task performance in a representative sample of the population here, so this weak criterion was not intended to select only those that successfully learned the association, but rather to exclude those that exhibited a very low willingness or ability to locate and consume the food. Across all groups, a total of eight females and four males did not proceed to the detour task trails. Prior to each trial, fish were gently guided with a net into the home compartment and the guillotine door was closed. The experimenter then placed a white plastic test plate (4 × 10 cm) with a green disc in the middle (diameter 1.5 cm) on the tank floor in the test compartment. A food reward was carefully placed on the green disc using a plastic pipette. For males, a single artemia was used, for females (which are much larger), we used three artemia. The guillotine door was then opened allowing the fish to swim into the test compartment and feed. The time to locate and eat the food reward was recorded for each fish using censored measurements. Specifically, we assessed whether the food item had been consumed at 1, 5, 10 and 20 min after opening the door, and at 20‐min intervals thereafter to a maximum of 140 min. This recording strategy allowed a single experimenter to collect data on 48 individuals simultaneously.

**FIGURE 2:**
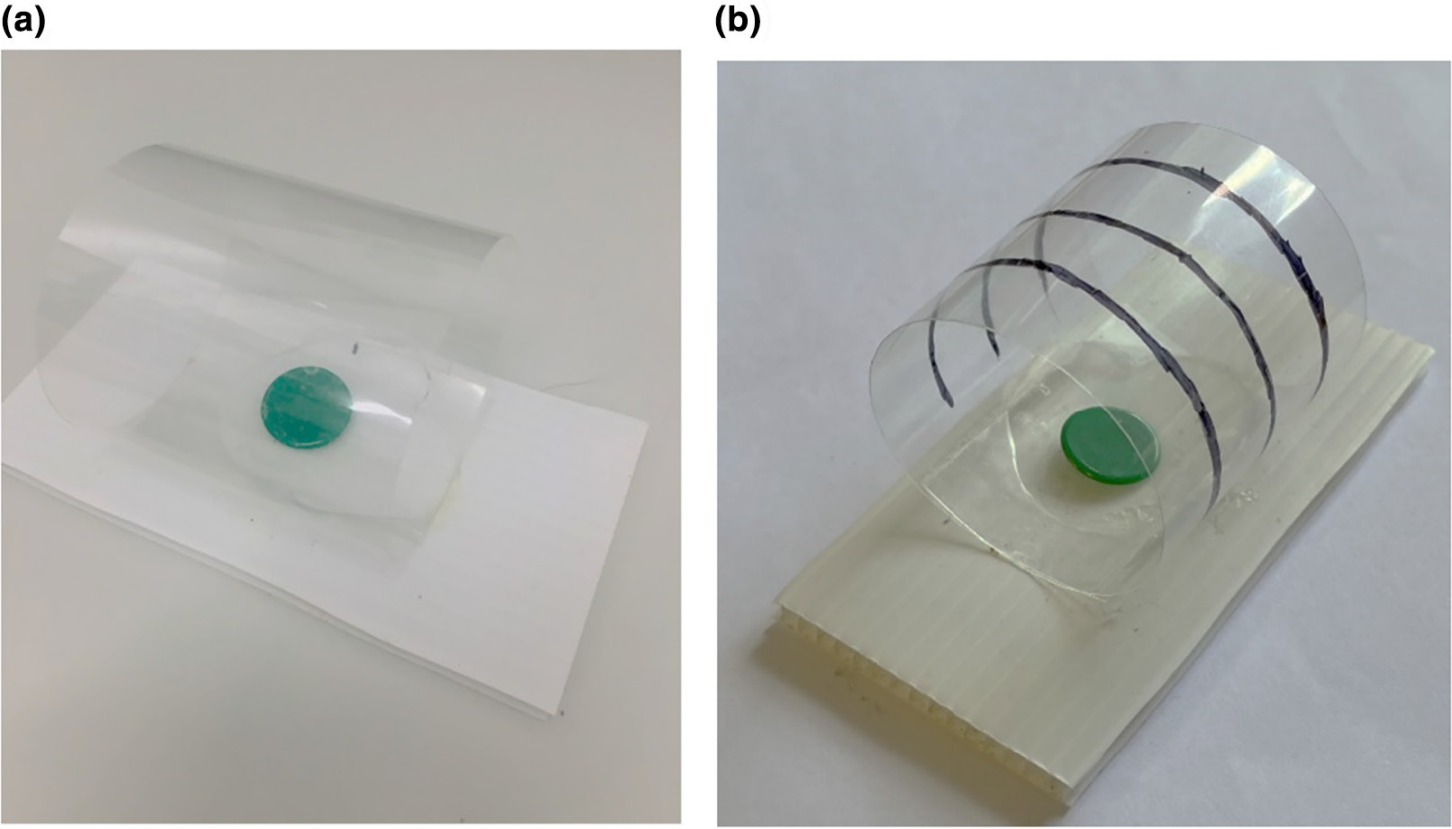
Photographs of the test plate used during the detour trials for (a) treatment 1, showing the (unmarked) transparent cylinder which represented low visual information, and for (b) treatment 2 which shows the cylinder with three black horizontal lines, used to represent high visual information. The photographs show the open ends of the cylinders in which fish could access to the food reward, in addition to the green plastic disc upon which the food reward was placed.

Fish that completed the training stage were then assayed three times in the detour task (once per day for three successive days). Detour task trials essentially repeated the training trials; fish were guided into the home compartment, the guillotine door closed and the food item was placed on the green disc. However, the green disc was now inside a transparent plastic cylinder (Figure 2). The green disc was visible, but fish were required to navigate around the barrier to gain access to the food reward from either end of the cylinder. In many detour task studies (Brandão et al., 2019; Lucon‐Xiccato et al., 2017; Macario et al., 2021), individuals are initially trained to extract food from an opaque cylinder before being presented with the transparent cylinder. We chose not to do this because (a) the initial training phase ensured that fish were motivated to swim towards the food and (b) an opaque phase complicates the interpretation of results. This is because rather than depending on inhibitory control (i.e., inhibiting the tendency to swim directly towards the food) successful performance could be explained by having learned during the opaque phase to swim towards the open ends of the cylinder (Ashton et al., 2018).

During the detour tasks, we recorded time to eat by assessing the presence/absence of the food item at 1, 5, 10 and 20 min after opening the door, and thereafter at 20‐min intervals for a further 2 h, then 60 min intervals for a further 5 h, and a final check at 24 h after opening the door. Unfortunately, severe constraints on researcher lab time (imposed by the Covid‐19 pandemic), precluded collection of more detailed behavioural data (e.g., number of redundant attempts to directly acquire the food reward through the barrier (Kabadayi et al., 2017; Santacà et al., 2019)). To test for GxE, fish were assigned to one of two treatments in the detour task; either a transparent (unmarked) cylinder with low visual information, or a cylinder marked with three black horizontal lines providing high visual information. Individuals experienced only one, randomly assigned treatment, but brood groups (and so sibships) were split across treatments.

### 2.4 Microsatellite genotyping and pedigree analysis

At the end of the experiment, both parents and offspring were euthanized by overdose of buffered MS‐222 and individually stored in 70% ethanol at −5°C. DNA was extracted from tail tissue and processed according to the protocol described in (Becher et al., 2002). Fish were genotyped fish at six autosomal microsatellite loci (see Table S1 for details) as described in (Becher et al., 2002) and (Bergero et al., 2019). Polymerase chain reaction (PCR) reaction conditions and the molecular protocol are described in full in Supplementary Information S2. Individual fish were genotyped using Genemapper® ID‐X software (Thermofisher Scientific, Waltham, MA, USA) by scoring genotypes across all six microsatellite markers. Pedigree reconstruction was enabled by the program COLONY 2.0.4.5 (http://www.zsl.org/science/software/COLONY), which reconstructs parental genotypes from offspring genotypes using maximum likelihood (Jones & Wang, 2010; Wang & Santure, 2009). The details of COLONY run parameters can be seen in Supplementary Information S3.

### 2.5 Statistical analyses

Data from both detour and training trials were analysed using linear mixed effect model fitted with ASReml‐R 4.1 (Butler et al., 2018; Gilmour et al., 2009) within R version 3.6.1 (R Development Core Team, 2008). We make the standard assumptions that random effects and residuals are normally distributed with means of zero and variances, to be estimated. All standard errors (SE) reported are estimated in ASReml‐R (using the delta method to obtain SE for derived parameters such as heritability that are functions of the variance components). *Time to eat* was used as the response variable in all models and was natural log‐transformed before being mean‐centred and scaled to standard deviation units (SDU). Log transformation improved the assumption of Gaussian error structure while rescaling to SDU eases interpretation of estimated variance components.

#### 2.5.1 Performance in detour task trials

For the detour task trials, we initially fitted a base model of:

$$\begin{array}{c} \text{Time to}\;\mathrm{eat}\sim \text{mean}+\text{treatment}+\text{stack}+\text{trial number}\\ \;+\mathrm{sex}+\text{block}+\text{brood tank}\;\left(\text{Model}\;A1\right) \end{array}$$

where *treatment* (1 = clear cylinder, 2 = marked cylinder), *stack* (denoting which of two aquaria stacks the fish was tested in) and *trial number* (i.e., the repeat number 1–3) were fitted as fixed factors. We also included *sex* (male, female) to control for any sexual dimorphism in mean inhibitory control. Since reversal learning tasks require inhibition of learned behaviours (Lai et al., 1995), reports that female guppies are faster than males to inhibit previously learned colour‐reward associations (Lucon‐Xiccato & Bisazza, 2014, 2017a) are suggestive of dimorphism. Note, however, that in the absence of specific a priori hypotheses about the evolution of sexual dimorphism, we do not model sex‐specific variance components (see description of random effects below). Conditional F‐statistics implemented in ASReml‐R were used to determine the significance of fixed effects. The base model also included a random effect of block (the set of up to 48 fish that were phenotyped simultaneously) and *brood tank*. The latter controls for any common environment effects arising from the early life housing environment.

A set of five further models (A2–A6) were then fitted to performance data from the detour trials. These models contain additional random effects as summarized in Table 1 to test for and estimate among‐individual variance in performance, including IxE (environmental sensitivity of among‐individual variance), before decomposing this into additive genetic (including GxE) and permanent environment components. Models A1–A6 were compared using Akaike information criterion (AIC) and, where nested, using likelihood ratio tests (LRT) and a standard hypothesis testing framework. Where LRT were applied to test a single variance component, we assumed twice the difference in log‐likelihood between full and reduced models is distributed as a 50:50 mix of ${\chi^{2}}_{0}$ and ${\chi^{2}}_{1}$ (Visscher, 2006). For all other situations, we (conservatively) set the degrees of freedom equal to the number of additional (co)variance components in the more complex model. To provide intuitive measures of effect size, we calculated the adjusted repeatability (R) of performance under Models A2 and A3, where R is conditional on fixed effects and represents the proportion of phenotypic variance explained by among‐individual differences in behavioural mean (Nakagawa & Schielzeth, 2010). Thus, *R* = *V*_I_/*V*_P_ where *V*_I_ is the among‐individual variance and *V*_P_ is the phenotypic variance conditional on fixed effects (i.e., *V*_P_ = *V*_I_ + *V*_B_ + *V*_BT_ + *V*_R_ where *V*_B_, *V*_BT_ and *V*_R_ are among‐ block, among‐brood tank and residual variances, respectively). Since model A3 allows for IxE, we estimate *R* for each level of the cylinder treatment. Under models A4 and A5, we similarly estimated adjusted heritabilities *h* ^2^ (where *h* ^2^ = *V*_A_/*V*_P,_ and is conditional on fixed effects). Note that in A5, which allows GxE, treatment‐specific estimates of *V*_A_ (additive genetic variance) and *V*_PE_ (permanent environment variance) are used as appropriate to estimate treatment‐specific *h* ^2^ and the cross‐treatment genetic correlation (*r*_G_). Note that, for the genetic part of the model, GxE would manifest as *V*_A1_ ≠ *V*_A2_ and/or *r*_G12_ < +1 (where subscripts 1 and 2 denote the clear and marked cylinder treatments, respectively).

**TABLE 1:** Summary of additional random effects included in expanded models of detour task performance (A2–A6) and training task performance (B2–B6).

Trials	Model	I	IxE	G	GxE	PE	PExE	IxT	GxT	PExT
Detour task	A2	x								
	A3	x	x							
	A4			x		x				
	A5			x	x	x	x			
	A6			x		x	x			
Training task	B2	x								
	B3			x		x				
	B4	x						x		
	B5			x		x			x	x
	B6			x		x				x

*Note*: Shaded cells denote inclusion of effects to estimate among individual (I), additive genetic (G) and permanent environment (PE) variance components as well as interactions with treatment (E) in the detour task and trial number (T) in training.

#### 2.5.2 Performance in the feeding training trials

The primary purpose of modelling performance in the training trials was to determine whether differences among individuals and/or genotypes were detectable at this stage of the experiment (i.e., before presentation of the IC task). We first fitted a base model identical to Model A1 above, but without the fixed effect of treatment:

$$\begin{array}{c} \text{Time to}\;\mathrm{eat}\sim \text{mean}+\text{stack}+\text{trial number}\\ \;+\mathrm{sex}+\text{block}+\text{brood tank}\;\left(\text{Model}\;B1\right) \end{array}$$

We then fitted and compared among further models (B2–B6) with additional random effects, as summarized in Table 1, to characterize among‐individual and genetic variance in training trial performance. Since all fish experienced the same conditions for the training trials, we did not model IxE or GxE across cylinder treatments. However, we did consider the possibility that there could be among‐individual and/or genotype variation in the pattern of any change in performance across the nine repeated training trials. We expect that, on average, time to obtain the food will decline across training trials (as individuals learn the association of the green disc with food). However, variation in this process could have implications for determining whether differences in training trial performance are sufficient to explain variation in detour task performance. This could occur if individuals (or genotypes) change performance ranking over the training period such that, for example, relative performance in early training trials poorly predicts performance at the end of the training periods. We therefore use ‘random slope’ models in which the effect of individual identity effect (B4) and/or additive genetic effect (B5) was modelled as a first order (linear) function of *trial number* (treated as a continuous covariate).

After finding variation in training trial performance was statistically supported (see results), we built bivariate mixed models to test the among‐individual and genetic relationships between performance in training trials and performance in the detour task. This allowed us to determine whether among‐individual (genetic) variance in training performance alone was sufficient to explain among‐individual (genetic) variance in detour test performance. Details of these model structure can be seen in full in Supplementary Information S6.

## 3 RESULTS

### 3.1 Detour task models

**FIGURE 3:**
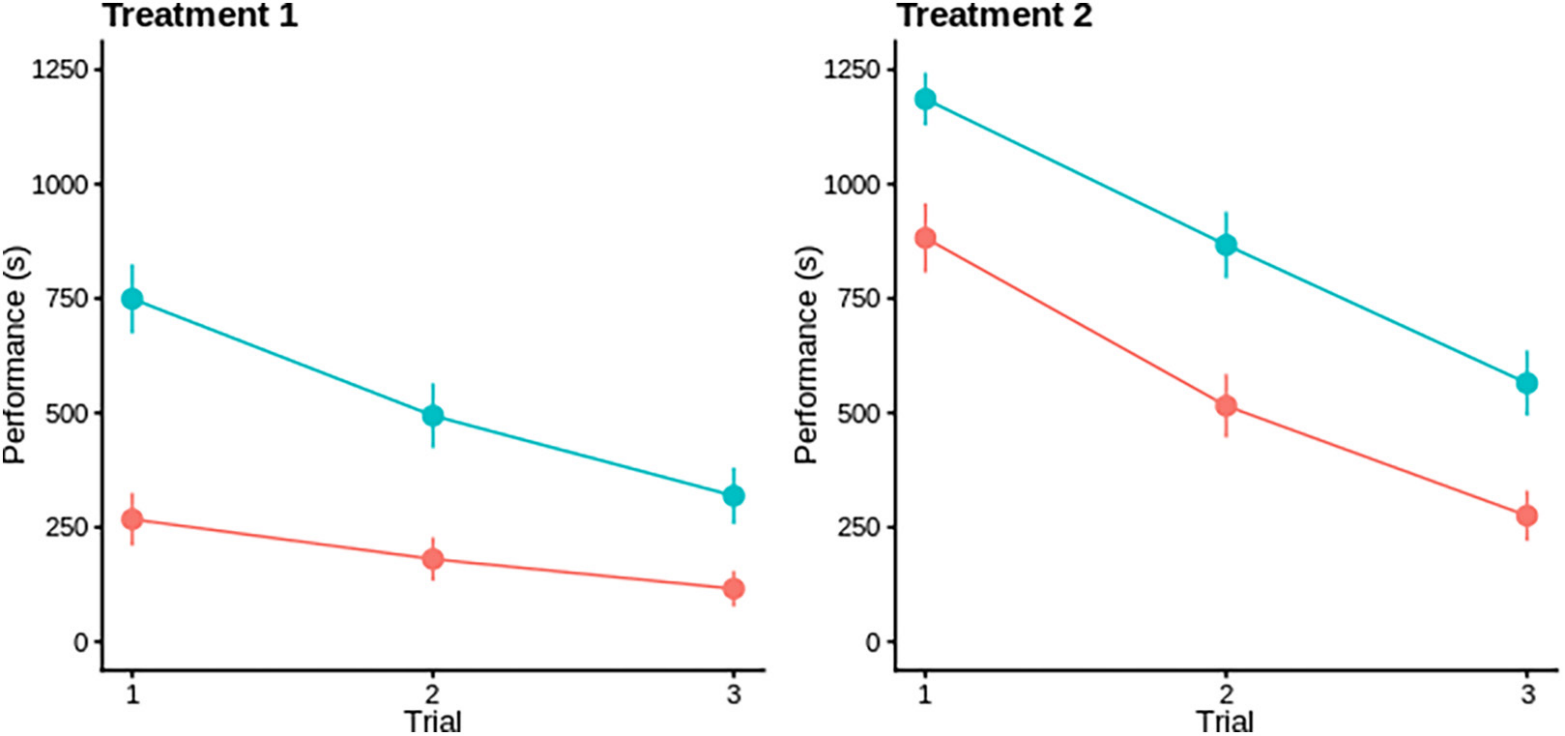
Plots of raw data of performance time across trials. Plots represent mean and standard errors for performance time in the detour task in treatment 1 (clear cylinder) and 2 (marked cylinder), across the three trials for males (blue) and females (red). Error bars represent mean and standard errors of performance time for individuals.

Plotting the raw data revealed an increase in average performance (i.e., decrease in time to eat) across repeated trials in both sexes and treatments. Females were consistently faster than males and both sexes obtained food more rapidly when presented with the clear cylinder (Figure 3). Univariate models of log‐transformed data in SDU confirmed the statistical significance of sexual dimorphism (Model A5: male vs. female *sex* coefficient ± SE = 0.615 ± 0.072, *F*_1,365.2_ = 73.4, *p* < 0.001) and improvement across repeats (Model A5: *trial 2* vs. *trial* 1 coefficient − 0.286 ± 0.047 and *trial* 3 vs. *trial 1* coefficient − 0.574 ± 0.04; *F*_2,713.5_ = 73.9, *p* < 0.001). They also supported the presence of significant plasticity in (mean) performance across the two treatment environments (Model A5; striped vs. clear *treatment* coefficient 0.646 ± 0.121, *F*_1,18.5_ = 28.3, *p* < 0.001). Note that these estimated fixed effects were very similar across models A1–A6 (see Supplementary Information Table S4 for all fixed effect results, including those not directly relevant to current hypotheses).

Model comparisons based on AIC and LRT provided strong support for the presence of among‐individual variation in performance (e.g., LRT Model A2 vs. Model A1; ${\chi^{2}}_{0,1}$ = 215, *p* < 0.001; Table 2). Under Model A2, performance in the detour task was highly repeatable (*R* = 0.467 ± 0.033; Table 3). Model A3 did not significantly improve the fit (LRT Model A3 vs. Model A2; ${\chi^{2}}_{1}$ = 0.124, *p* = 0.725) and yielded treatment specific estimates of among‐individual variance that were very similar (Treatment 1 (*V*_I_ = 0.372 ± 0.056); Treatment 2 (*V*_I_ = 0.343 ± 0.052); Table 3). Thus, there is strong evidence of among‐individual variance and population level plasticity across the treatments, but not for IxE.

**TABLE 2:** LRT and AIC model comparison—detour task.

Model	AIC	LnL	LRT comparison	Test for	*Χ* ^2^	DF	*p*
A1	799.719	−396.860	–	–	–	–	–
A2	587.178	−289.589	2 versus 1	V_I_	214.541	0,1	<0.001
A3	589.054	−289.527	3 versus 2	IxE	0.124	1	0.725
A4	589.178	−289.589	4 versus 2	V_A_	0	0,1	0.498
A5	586.524	−285.262	–	–	–	–	–
A6	591.054	−289.527	6 versus 5	GxE	8.53	2	0.014

*Note*: AIC values and likelihood (LnL) ratio test comparisons across univariate models for among‐individual (*V*_I_) and genetic (*V*_A_) variation in performance in the detour task trials.

**TABLE 3:** Estimated variance components and derived parameters for the six models of performance in the detour task trials.

Parameter	Model					
	A1 null model	A2 phenotypic model	A3 IxE model	A4 animal model	A5 full GxE model	A6 animal model with PxE
*V*_B_	0.018 (0.013)	0.016 (0.014)	0.015 (0.013)	0.016 (0.014)	0.013 (0.01)	0.015 (0.013)
*V*_BT_	0.013 (0.012)	0.000 (−)	0.000 (−)	0.000 (−)	0.000 (−)	0.000 (−)
*V*_R_	0.730 (0.032)	0.392 (0.021)	0.392 (0.021)	0.392 (0.021)	0.392 (0.021)	0.392 (0.021)
*V*_I_	–	0.357 (0.038)	0.372 (0.056)_1_ 0.343 (0.052)_2_	–	–	–
*V*_A_	–	–	–	0.000 (−)	0.253 (0.140)_1_ 0.000 (−)_2_	0.000 (−)
*V*_PE_	–	–	–	0.357 (0.038)	0.174 (0.092)_1_ 0.336 (0.051)_2_	0.371 (0.056)_1_ 0.343 (0.051)_2_
*r*_G_	–	–	–	–	−0.999 (−)	–
*R*	–	0.467 (0.033)	0.477 (0.043)_1_ 0.458 (0.042)_2_	–	–	–
*h* ^2^	–	–	–	0.000 (−)	0.304 (0.146)_1_ 0.000 (−)_2_	0.000 (−)
pe^2^	–	–	–	0.467 (0.033)	0.209 (0.119)_1_ 0.454 (0.042)_2_	0.477 (0.043)_1_ 0.458 (0.042)_2_

*Note*: Subscripts denote block (B), brood tank (BT), residual (R), individual (I), permanent environment (PE) and additive genetic (A) components of variance. Also shown, where applicable are corresponding estimates of repeatability (R), heritability (*h* ^2^), the intraclass correlation corresponding to *V*_PE_ (denoted pe^2^) and the cross‐treatment genetic correlation (*r*_G_). Parameter estimates specific to treatment 1 (clear cylinder) and 2 (marked cylinder) are denoted with subscripts and associated standard errors from each model are provided in parentheses where available. Note that variances were constrained to be positive and correlations between −1 and +1. Where parameters are fixed at boundary conditions no SE is estimated.

Statistical support for genetic contributions to variance in detour task performance is somewhat equivocal. On one hand, the animal model was not a better fit than the simple repeated measures mixed model (LRT comparison of Models A4 and A2; Table 2) and in fact the V_A_ estimate was bound to zero in the former. On the other hand, LRT comparison suggested Model A5 was a significant improvement on Model A6 (which allowed no GxE and constrained constant V_A_ across treatments; ${\chi^{2}}_{2}$ = 8.53, *p* = 0.014). Furthermore, the model with the lowest AIC was Model A5 (GxE) suggesting that, to the extent genes do matter, so does GxE. Model A5 certainly provides our best estimate of genetic parameters and indicates the presence of moderate V_A_ under treatment 1, while V_A_ in treatment 2 was bound to zero (Model A5; treatment 1, *V*_A_ = 0.253 ± 0.140, *h* ^2^ = 0.304 ± 0.146, Table 3). The cross‐treatment genetic correlation was not only estimated as negative but also bound to the edge of permissible parameter space (*r*_G.treatment1.2_ = −0.999, no standard error estimated). Note also that while random effects of *block* and *brood tank* were included in all models, associated variances were consistently non‐significant and low (with *V*_BT_ estimates bound to zero in most models; Table 3).

### 3.2 Training trial models

**FIGURE 4:**
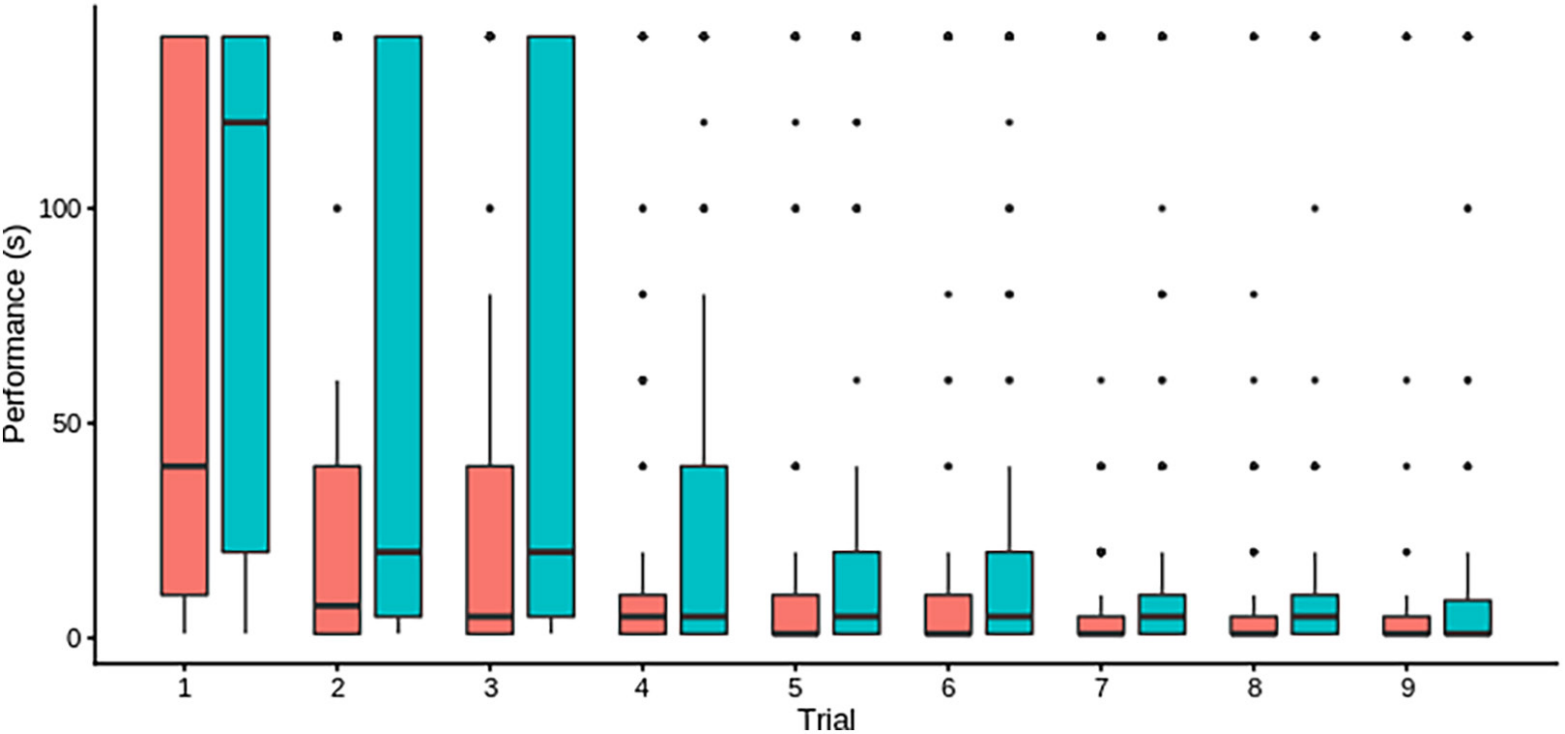
Boxplot of data distributions for performance (time to locate and eat the food reward) in the nine training trials for males (blue) and females (red). Horizontal lines within box correspond to behavioural medians, box boundaries correspond to first and third quartiles. When present, whiskers correspond to 10th and 90th percentiles, and points correspond to outliers.

Based on observations alone, fish tended to swim straight towards the food reward by the end of the training phase. This observation was supported by a strong pattern of improvement in average performance (i.e., decreases in time to obtain food) across training trials when plotting the raw data, with males again slower on average (Figure 4). Formal modelling confirmed the statistical significance of *sex* (Model B3: male vs. female *sex* coefficient ± SE = 0.421 ± 0.086, *F*_1,281_ = 23.8, *p* < 0.001) and *trial number* effect (*F*_8,2373_ = 184, *p* < 0.001 with coefficients becoming more negative as *trial number* increases; Table S4). In contrast to the detour task itself, we detect an effect of *stack* on time to eat in training, with fish in stack B being slower on average than those in stack A (Model B3: coefficient = 0.243 ± 0.087, *F*_1,282.7_ = 7.85, *p* = 0.005). Estimated fixed effect estimates were very similar across models B1‐B6 (presented in full in Table S4).

Model comparisons supported the presence of among‐individual variation in *time to eat* during training trials (LRT Model B2 vs. Model B1; ${\chi^{2}}_{0,1}$ = 1142, *p* < 0.001, Table 4) with Model B2 yielding an estimate of R = 0.485 ± 0.033 (Table S5). Model B3 (animal model) was a significant improvement on this (LRT Model B3 vs. Model B2; ${\chi^{2}}_{0,1}$ = 4.99, *p* = 0.013) providing evidence of genetic variance (*h* ^2^ = 0.170 ± 0.88; Table S5). The random regression modelling also supported among‐individual variation in the rate of improvement across training trials (IxT; LRT Model B4 vs. Model B2; ${\chi^{2}}_{0,2}$ = 117, *p* < 0.001, Table 4). Among‐individual variance in intercepts (int) and slope (slp) were estimated as *V*_I_int_ = 0.581 ± 0.064 and *V*_I_slp_ = 0.007 ± 0.001, respectively. The among‐individual intercept–slope correlation was estimated as *r*_I_int_slp_ = − 0.575 ± 0.057. Here, the negative sign means those individuals with worst initial performance (high intercepts) tended to improve most (more negative slopes) across repeated training trial. However, Model B5 (GxT model) was not a significant improvement on Model B6 (IxT present but arising from non‐genetic PExT effects only; LRT Model B5 vs. Model B6; ${\chi^{2}}_{2}$ = 1.347, *p* = 0.509). Thus, we find no statistical support for the presence of GxT for training trial performance across repeated *trial number*. Estimated variance components associated with *block* and *brood tank* effects were low and non‐significant across all six model formulations (Table S5).

**TABLE 4:** LRT and AIC model comparison—training trials.

Model	AIC	LnL	LRT comparison	Test for	*Χ* ^2^	DF	*p*
B1	1803.996	−898.998	–	–	–	–	–
B2	663.521	−327.761	2 versus 1	V_I_	1142.475	0,1	<0.001
B3	660.528	−325.264	3 versus 2	V_A_	4.994	0,1	0.013
B4	550.762	−269.381	4 versus 2	IxE	116.760	2	<0.001
B5	603.238	−266.116	5 versus 4	–	–	–	–
B6	547.580	−266.789	6 versus 5	GxE	1.347	2	0.509

*Note*: AIC values and likelihood ratio test comparisons across univariate models for performance in the training trials.

There is thus strong statistical support for IxT (but not GxT) in training trial performance, where T is the ‘environmental’ axis of experience as measured by *trial number*. Since the consequences of this for current purposes are not immediately apparent, we projected the among‐individual intercept‐slope covariance matrix to a ‘character state’ view [following e.g., (Nussey et al., 2007). This simply transforms the slope‐intercept (co)variance structure to an estimate of the among‐individual covariance matrix for the nine *trial number* specific performances. This transformation shows that, while IxT is statistically significant, its magnitude is insufficient to cause much change in V_I_ with *trial number* or to disrupt the uniformly positive among‐individual correlation structure (Table S6). This means individual rank ordering of performance is largely maintained across the training period (i.e., there is not much reaction norm crossing). Due to this result, we elected to revert to a random intercepts approach for this trait in the bivariate model.

### 3.3 Bivariate analysis of detour task and training trial performance

The bivariate model yielded an estimate of the among‐individual covariance structure (**ID**) that revealed positive relationships between performance in treatment‐specific detour tasks and training trials (comparison of the full model to one in which all among‐individual between trait covariances are fixed to zero; ${\chi^{2}}_{3}$ = 33.389, *p* < 0.001). Estimated among‐individual correlations (± SE) were 0.491 ± 0.089 in treatment 1 (clear cylinder) and 0.306 ± 0.097 in treatment 2 (marked cylinder; Table 5). A strong positive correlation between individual performance in the two treatment specific detour task traits was also estimated (*r*_ind.treatment1.2_ = 0.816 ± 0.610; Table 5). This parameter is, strictly speaking, identifiable in the model. However, as no individual experienced both treatments, the data contain very little information for its estimation (reflected in the large SE), and we do not interpret it further.

**TABLE 5:** Bivariate mixed model output.

	Training trials	Detour task	
		Treatment 1	Treatment 2
ID Matrix			
Training Trials	**0.379 (0.037**)	**0.491 (0.089)**	**0.306 (0.097)**
Detour Task–Treatment 1	**0.187 (0.040)**	**0.376 (0.037)**	0.816 (0.607)
Detour Task–Treatment 2	**0.110 (0.038)**	0.293 (0.220)	**0.343 (0.052)**
G Matrix			
Training Trials	0.125 (0.075)	0.226 (0.365)	−0.999 (−)
Detour Task–Treatment 1	–	**0.299 (0.145)**	−0.999 (−)
Detour Task–Treatment 2	–	–	0.000 (−)
PE Matrix			
Training Trials	**0.318 (0.056)**	**0.696 (0.247)**	**0.364 (0.106)**
Detour Task–Treatment 1	–	0.157 (0.093)	0.999 (−)
Detour Task–Treatment 2	–	–	**0.335 (0.051)**

*Note*: These models tested the among‐individual and genetic relationships between performance in training trials and performance in the detour task. Shown are the variance–covariance–correlation matrix estimates from among Individual (ID) and genetic (G) bivariate mixed models (including the permanent environment (PE) matrix). Estimated variances are shown on the diagonal (dark grey shading), (covariances are not shown as back‐calculating them becomes problematic with variances bound to zero and/or correlations at ±1). Standard errors are shown in parentheses where available, and bold font denotes nominally significant estimates assuming approximate 95% CI of ±1.96 SE.

Extending the bivariate model to partition **ID** into **G** and **PE** added relatively little additional insight. Estimates of genetic variance for training trial performance (*V*_A_ = 0.124 ± 0.075) and detour task performance under treatment 1 (*V*_A_ = 0.290 ± 0.145) were similar to estimates from univariate models. The genetic correlation between these was relatively weak (*r*_G_ = 0.227 ± 0.364). The estimated genetic variance for detour task performance under treatment 2 was again bound to zero, and genetic correlation estimates with this trait were bound to −1 (but are not sensibly interpreted in the absence of detectable genetic variance; Table 5).

## 4 DISCUSSION

In this study, we sought to characterize among‐individual and genetic variation in inhibitory control, using performance in a detour task as an observable proxy for the latent ability to inhibit prepotent behaviour (Diamond, 1990; Kabadayi et al., 2018; MacLean et al., 2014). We find individuals vary considerably in detour task performance and thus, subject to assumptions discussed below, conclude they are likely to differ in inhibitory control. We also find strong evidence of plasticity in average performance across treatments though the direction is perhaps counterintuitive; fish took longer on average to obtain the food item when visual information was increased by adding lines to the clear cylinder. Support for genetic contributions to among‐individual variance was somewhat mixed. Although we find statistical support for a GxE interaction, this actually results in heritable variation being evident only in one treatment (the clear cylinder). We also show that individuals differ in performance during the training period used to establish the cue‐reward association. Training performance positively predicts, but is insufficient to fully explain, variation in the detour task. In what follows, we discuss these results in the context of understanding variation in, and evolution of, animal cognition. However, we also highlight caveats and assumptions arising from the use of performance in the detour task as a proxy for the latent cognitive trait of inhibitory control and consider alternative explanations for the observed results.

### 4.1 Among‐individual and genetic variance in detour task performance

Guppies in our study show consistent among‐individual variation in detour task performance measured as time to navigate a barrier and obtain a food reward. Repeatability is moderately high (e.g., 47% averaged across the treatments) in comparison to estimates for other cognitive trait measures in animals [see (Cauchoix et al., 2018) for a review]. Accepting that the task is a valid proxy of inhibitory control (an assumption explored further below), this result is consistent with prior studies using similar experimental paradigms in guppies (Lucon‐Xiccato & Bertolucci, 2019; Lucon‐Xiccato, Bisazza, & Bertolucci, 2020; Macario et al., 2021). We also see learning (i.e., fish improve over successive trials) and sexual dimorphism (i.e., females out‐performing males) that recapitulate findings from previous studies [see also (Triki et al., 2022)]. Though we did not directly observe behaviour here, others have noted males are more persistent in trying to pass through a transparent barrier (Lucon‐Xiccato & Bisazza, 2017a). Additionally, several studies using reversal learning tasks have shown males present lower cognitive flexibility than females when a previously learned response becomes inappropriate (Brandão et al., 2019; Lucon‐Xiccato & Bisazza, 2014). Speculatively, dimorphism of inhibitory control might actually be adaptive and reflect sex‐specific reward systems since, for example, persistent male harassment of females can be rewarded by higher rates of copulation (Endler, 1984; MaGurran & Seghers, 1994). However, it is possible that the sex differences in performance we observe could arise from differences in reinforcement of the cue‐reward association, rather than in learning ability per se.

We did not find evidence of a significant genetic contribution to among‐individual variation when using the standard animal model that assumes genetic parameters are constant across treatments. This result contrasts with reports of moderate *h* ^2^ for inhibitory control in humans and other animals (Cervantes et al., 2013; Friedman et al., 2008; Gnanadesikan et al., 2020; Schachar et al., 2011). However, when modelling genotype‐by‐environment interaction (GxE), our analyses suggest context‐specific moderate heritability (26.3%) that is limited to the clear cylinder treatment. From an evolutionary perspective, the implication of this result is that whether inhibitory control will respond to directional selection at all may depend on environmental conditions.

GxE not only implies the presence of environment‐specific genetic variance but can also be understood as genetic variance for phenotypic plasticity (Hill & Mulder, 2010; Pigliucci, 2001). Viewing our results from this perspective presents some complementary insights. Although our experimental design precludes direct observation of individual‐level plastic responses of performance to treatment, we know plasticity occurs because average detour task performance differs between clear and marked cylinders. While naïvely expecting the marked cylinder to provide more visual information and facilitate faster location of the food reward (Juszczak & Miller, 2016; Santos et al., 1999) we actually found the opposite. It is possible that the marks on the more visible cylinder distracted fish from observing and locating the food reward, or they presented as a mildly aversive stimulus [e.g., eliciting neophobia or predator avoidance behaviour (113)]. Irrespective, an important point is that GxE for performance detected in the detour task need not imply GxE for the latent trait of inhibitory control if observed time to eat also depends on other traits (e.g., motivation, neophobia) that are also genetically variable in at least some environments.

Time to complete a detour task is a widely used proxy for inhibitory control [although additional behaviours are often measured with it (Lucon‐Xiccato et al., 2017; Macario et al., 2021). We suggest that here—and in most cognitive studies—it is risky to uncritically attribute all variation to a single latent trait. For instance, one recent study concluded that variation in problem solving by great tits could be fully explained by differences in motivation, selective attention, and prior experience without any underlying cognitive variation per se (Cooke et al., 2021). In our work, more detailed behavioural observations [e.g., number of redundant attempts to directly acquire the food reward through the barrier (Friedman et al., 2008; MaGurran & Seghers, 1994)] would have been valuable to further validate the assumed link between *time to eat* and inhibitory control. Unfortunately, pandemic restrictions precluded such behavioural phenotyping. We also note that our design differs from many detour tasks in that subjects were not initially trained gain access to food from either end of an opaque cylinder. While this difference complicates comparisons to other studies, our initial training was designed to ensure that participating guppies were motivated to swim directly to the food. Successful performance in the detour task is therefore likely to reflect the ability to inhibit this prepotent response (given that the guppies could still see the food through the cylinder). Nevertheless, we fully acknowledge that completing our detour task may involve additional cognitive processes (e.g., working memory, route planning).

Moreover, among‐individual and/or genetic variance may stem from other aspects of behavioural traits [e.g., exploration (Kabadayi et al., 2018), flexibility (Coomes et al., 2022), persistence (Bensky & Bell, 2020), or ‘state’ (e.g., physiological condition, motivation)] that could not be fully experimentally controlled. If so, variance in detour task performance will depend not just on variance in contributing traits, but also in covariance between them. For instance, in the hypothetical case that *p = ic + s* (where *p* is detour task performance, *ic* is inhibitory control, and s is state), then *V*_A(p)_ = *V*_A(ic)_ + *V*_A(s)_ + 2COV_A(ic,s)_. Thus, a lack of genetic variance for performance in the marked cylinder treatment could plausibly arise from a context‐specific negative genetic covariance between inhibitory control and some other trait(s). Though entirely speculative in the present case, context‐specific covariance structure between inhibitory control and foraging flexibility has been reported in great tits (Coomes et al., 2022). We also note that, lack of detectable genetic variance in some contexts could arise from genotype‐by‐sex (GxS) interactions on inhibitory control (e.g., given negative cross‐sex genetic covariance) and/or GxExS (i.e., sex‐specific heritability of plastic responses to context) that were not modelled here. Additional post hoc modelling (not shown) provided no evidence of significant GxS on detour task performance although we note power may be limited and suggest further exploration of sex‐limited genetic variance for inhibitory control (and other cognitive traits) is warranted.

### 4.2 Variation in training performance

Several additional conclusions emerge from our analysis of the performance in the training portion of the study. Females are again faster to eat than males, and both sexes improve average performance over successive trials, suggesting that training of the colour signal‐food reward association was successful. While not a specific objective of our study, this result contributes to the growing literature on associative learning in guppies (Kniel et al., 2020; Lucon‐Xiccato & Bisazza, 2017b; Trompf & Brown, 2014). Individual fish also vary, both in training trial performance overall (averaged across trials) and in the rate of improvement, and in learning [following e.g., (Langley et al., 2020)] across trials. There is a significant genetic contribution to the former (*h* ^2^ = 17%), but not the latter (i.e., no significant GxT). This implies average performance is heritable while learning is not, although some caution is warranted in drawing this conclusion. First, the ‘fastest learners’ (i.e., those fish with steepest negative slopes) still tend to be the poorer performers over all trials (i.e., learning fast is associated with having a lot of ‘room for improvement’). Second, IxT for *time to eat* in training is consistent with variation in learning ability, but could also arise without cognitive differences if, for example, exploratory tendency (Boogert et al., 2006; Bousquet et al., 2015; Zidar et al., 2018) or motivation (van Horik & Madden, 2016) changes across trial number at rates that differ among fish. Excluding these possibilities is experimentally challenging, particularly if associative learning is correlated with personality (e.g., exploration, boldness) which has been reported in some fish and bird studies (DePasquale et al., 2014; Guillette et al., 2009; Quinn et al., 2016).

Although variation in training performance is thus interesting in its own right, our primary rationale for analysing this data was to help validate the detour task as being informative for inhibitory control. This position would be difficult to justify if variation in time to eat among‐individuals (and/or genotypes) without the cylinder present was actually sufficient to explain variation when it was. Here, we find individual performance in training positively predicts detour task performance (in both cylinder treatments) but also that the corresponding among‐individual correlations are significantly less than +1 (based on their approximate 95% CI). From this, we conclude that that factors causing among‐individual variation in training contribute to, but are insufficient to fully explain, differences in detour task performance. More quantitatively, the estimated covariance structure in **ID** implies that 75% of among‐individual variance in the detour task in treatment 1, and 90% in treatment 2, is statistically independent of training performance [based on calculation of conditional variances following (Hansen & Houle, 2008)]. This result strengthens our assumption that the detour task is informative for inhibitory control, even if personality, motivation, associative learning and/or other unmeasured factors may also contribute to performance in both training and detour task trials. Again, we note that such latent factors can themselves be correlated with each other and with inhibitory control (Ashton et al., 2018; Langley et al., 2020; Lucon‐Xiccato, Montalbano, & Bertolucci, 2020) such that disentangling their contributions to variable performance is challenging.

### 4.3 Summary

In conclusion, we find evidence of among‐individual variation in inhibitory control in guppies. Our results suggest GxE occurs such that heritable variation is present when a standard detour testing paradigm is applied, but not when additional visual information is provided in the form of a marked cylinder. This implies that genetic variance in, and so potential for further adaptive evolution of, inhibitory control may be highly sensitive to environmental contexts. An alternative view of the same phenomenon is that plasticity in detour task performance, and so (with some assumptions) inhibitory control, is genetically variable. We also find that an individual's performance in training trials used to create a food‐cue association positively predicts—but is insufficient to completely explain—its performance in the detour task. As such, we conclude that the detour task is effectively capturing distinct cognitive processes (i.e., inhibitory control), although other cognitive and/or personality traits very likely contributing to performance as well.

## AUTHOR CONTRIBUTIONS

**Pamela M Prentice:** Conceptualization (lead); data curation (lead); formal analysis (equal); investigation (lead); methodology (equal); writing – original draft (lead). **Alexander Thornton:** Conceptualization (supporting); supervision (supporting); writing – review and editing (equal). **Niclas Kolm:** Conceptualization (supporting); methodology (equal); writing – review and editing (equal). **A. J. Wilson:** Conceptualization (supporting); formal analysis (supporting); supervision (lead); writing – review and editing (equal).

## CONFLICT OF INTEREST

The authors have no conflict of interest to declare.

### PEER REVIEW

The peer review history for this article is available at https://www.webofscience.com/api/gateway/wos/peer‐review/10.1111/jeb.14241.

## Supporting information


Data S1

## Data Availability

The data that support the findings of this study are openly available in Dryad at https://doi.org/10.5061/dryad.66t1g1k71.
