# Supplementary material for: Genetic and context‐specific effects on individual inhibitory control performance in the guppy (Poecilia reticulata)
Source: J Evol Biol. 2023 Nov 2;36(12):1796–810. doi: 10.1111/jeb.14241 (PMC10947024; doi:10.1111/jeb.14241)
Supplement: Supplementary file 1 — Data S1 [file JEB-36-1796-s001.docx]

**Supplementary Information**

**Table S1**. Characterization of six polymorphic *Poecilia reticulata* microsatellite loci

| Primer | Forward Sequence | Reverse Sequence | T_a_ | Allele Range | Reference |
| --- | --- | --- | --- | --- | --- |
| Pr39 | GGTAAGGACTGATGAATAGCTTG | TTAGGGCCGTGTCTTTTG | 55 | 157 - 175 | (1) |
| Pr40 | AGGTGGGATGAAGTCGTG | ACCAGAGCCAAACAACAAATC |  | 232 - 424 |  |
| Pr80 | GTACGAACTCTCTCGCAA | TGTGGTTTAGGTTGGACTGGG |  | 124 - 212 |  |
| Pr92 | ACCCTGTGCAGAGCAAAGAC | TGGGCTGCTTTGTGAAGT |  | 156 - 174 |  |
| Ag78 | ATATCCACTCTCCACTCTGCATGC | CCACTCATTGTCTTGAGACCCTGC | 55 | 216 - 260 | (2) |
| Ag155 | CTCTGCTGGAGCGAATGGTCAAC | GCATAACAATCTGAGCGGATCATTCC |  | 364 - 432 |  |

Ta, annealing temperature

**S2: Molecular protocol**

At the end of the breeding stage, remaining parental generation individuals were euthanised by overdose of buffered MS-222 and individually stored in 70% ethanol at -5°C. All offspring individuals were similarly euthanised at the end of behavioural data collection. For all individuals, DNA was extracted from tail tissue and processed according to the protocol described in (1). We then genotyped fish at 6 autosomal microsatellite loci (Table S1 for details) as described in (1) and (2). Polymerase chain reaction (PCR) reaction conditions were as follows: 8.3 μL GoTaq Mastermix (Promega), 0.2 μL each of fluorescently-labelled forward and reverse primer, 7.8 μL nuclease free-water and 2.5 μL DNA. The PCR programme consisted of 3 minutes at 90 °C, then 30 cycles of 30 seconds at 90 °C, 20 seconds at 55 °C and 30 seconds at 72 °C, before a final extension period at 72 °C for 5 minutes. A random selection of individual amplifications across all 6 primers were visualized on 2% agarose gel to confirm successful amplification. Following this, individual PCR products were separated by capillary electrophoresis in AB 3500 Genetic Analyzer (Thermofisher Scientific, Waltham, MA, USA).

We genotyped the parental and offspring generations using the Genemapper ® ID-X software (Thermofisher Scientific, Waltham, MA, USA) by scoring individual genotypes across all 6 microsatellite markers using GENEMAPPER 3.7. Population allele frequencies were estimated in CERVUS 3.0 using the genotypes of the offspring generation across the six breeding groups. Individuals were assigned to full- and half-sibships using the program COLONY 2.0.4.5 (http://www.zsl.org/science/software/COLONY), which reconstructs parental genotypes from offspring genotypes using maximum likelihood (3,4). Colony runs were programmed so that (half) sibships were assigned within the offspring of each breeding group separately (recognising that relatedness across groups is assumed to be zero) and that the ‘full likelihood structure‘ was then obtained for each group and combined across groups to give an overall pedigree structure. The details of COLONY run parameters can be seen in S3.

**S3: COLONY Parameters**

We calculated heritability of performance in the detour task and the training trials (and their genetic correlation) using a pedigree reconstructed from parent-offspring and sibship relatedness calculated using the COLONY software ([http://www.zsl.org/science/software/COLONY](http://www.zsl.org/science/software/colony)). COLONY simultaneously determines sibships and parentage by using maximum likelihood analysis, including a full-likelihood method and a pair-wise-likelihood method. We opted for the full likelihood method which is more computationally intense than the pair wise method but also more accurate, and as our dataset was relatively small, this was the most logical and conservative method. The offspring from each of our six breeding groups were run through COLONY independently. This increased accuracy of parentage assignment as parents only contributed to their own distinct breeding group.

We set mating system parameters for male and female polygamy, in a dioecious, diploid population, without the possibility for inbreeding. The run was set for “long” with a full likelihood analysis method set at medium precision, and no sib-ship priors. We informed our COLONY runs with allele frequencies estimated from the combined offspring population across all breeding groups, calculated within Cervus (CERVUS 3.0; Kalinowski et al., 2007). The combined error and mutation rate for genotypes was set at 0.05 to ensure flexibility in parental assignments, as suggested in the documentation for COLONY for a similar sample with six microsatellite loci (6).The probability of a parent being included in the candidate genotypes within each breeding group was set at 75%, and a genotyping error rate of 0.5% was used in all constructions. Only parent–offspring relationships with probability p-value less than 0.05 were retained and used within the pedigree (3,7).

**Table S4a: Univariate model fixed effects - detour task.** Fixed effect estimates (with standard errors in parentheses) from Model A5 of the detour task data. Intercept represents estimate at Trial number 1, sex Female, Treatment 1 and Stack A

| Model |  | Fixed effect | Effect size (SE) | DF | F | P |
| --- | --- | --- | --- | --- | --- | --- |
| A5 | Mean | Intercept | -0.447 (0.130) | 1, 15.4 | 10.060 | 0.006 |
|  |  | Trial number (2) | -0.286 (0.047) | 2, 713.5 | 73.890 | <0.001 |
|  |  | Trial number (3) | -0.574 (0.047) |  |  |  |
|  |  | Sex (Male) | 0.615 (0.071) | 1, 365.2 | 73.350 | <0.001 |
|  |  | Treatment (2) | 0.646 (0.121) | 1, 381.6 | 28.340 | <0.001 |
|  |  | Stack (B) | 0.198 (0.072) | 1, 380.2 | 7.591 | 0.006 |

**Table S4b:** **Univariate model fixed effect – training trials**. Fixed effect estimates (with standard errors in parentheses) from Model B3 of the training trails. Intercept represents estimate at Trial number 1, sex Female and Stack A

| Model |  | Fixed effect | Effect size (SE) | DF | F | P |
| --- | --- | --- | --- | --- | --- | --- |
| B3 | Mean | Intercept | 0.622 (0.118) | 1, 12.6 | 0.000 | 0.998 |
|  |  | Trial number (2) | -0.574 (0.048) | 8, 2372.7 | 183.8 | <0.001 |
|  |  | Trial number (3) | -0.694 (0.048) |  |  |  |
|  |  | Trial number (4) | -0.944 (0.048) |  |  |  |
|  |  | Trial number (5) | -1.140 (0.048) |  |  |  |
|  |  | Trial number (6) | -1.143 (0.048) |  |  |  |
|  |  | Trial number (7) | -1.351 (0.048) |  |  |  |
|  |  | Trial number (8) | -1.340 (0.048) |  |  |  |
|  |  | Trial number (9) | -1.430 (0.048) |  |  |  |
|  |  | Sex (Male) | 0.421 (0.086) | 1, 281 | 23.840 | <0.001 |
|  |  | Stack (B) | 0.243 (0.087) | 1, 282.7 | 7853 | 0.005 |

**Table S5:** Estimated variance components and derived parameters for the six models of performance in the training trials. Subscripts denote block (B), brood tank (BT), residual (R), individual (I), permanent environment (PE) and additive genetic (A) components of variance. Also shown is variance in slopes (V_slp_), at individual (V_I.slp_)_,_ additive (V_A.slp_) and permanent environment (V_PE.slp_) levels. Also, the correlation estimate between intercepts and slopes (r) at these levels. In addition, we show the familiar measures of repeatability (R) and heritability (h^2^) of mean performance across all models. Values in parentheses indicate approximate standard errors. Nominally significant estimates assuming approximate 95% CI of ± 1.96SE are denoted with (*) for P<0.05 and (**) for P<0.001.

| Parameter | Model | | |  | | |
| --- | --- | --- | --- | --- | --- | --- |
|  | B1  null model | B2  Phenotypic model | B3  Animal model | B4  Random regression model (IxE) | B5  Random regression animal model (GxE) | B6  Random regression animal model (no GxE) |
| V_B_ | 0.016 (0.018) | 0.023 (0.021) | 0.005 (0.01) | 0.023 (0.022) | 0.000 (0.012) | 0.005 (0.018) |
| V_BT_ | 0.110 (0.029) | 0.035 (0.023) | 0.000 (-) | 0.034 (0.023) | 0.000 (-) | 0.000 (-) |
| V_R_ | 0.677 (0.019) | 0.349 (0.010) | 0.350 (0.010) | 0.299 (0.009) | 0.299 (0.001) | 0.299 (0.009) |
|  |  |  |  |  |  |  |
| V_I_ | - | 0.384 (0.023)** | - | 0.581 (0.064) | - | - |
| V_I.slp_ | - | - | - | 0.007 (0.001) | - | - |
| r_I_ | - | - | - | -0.575 (0.057) | - | - |
|  |  |  |  |  |  |  |
| V_A_ | - | - | 0.136 (0.056)* | - | 0.014 (0.093) | 0.135 (0.075) |
| V_A.slp_ | - | - | - |  | 0.000 (0.001) | - |
| r_G_ | - | - | - | - | -0.195 (0.643) | - |
|  |  |  |  |  |  |  |
| V_PE_ | - | - | 0.309 (0.076) | - | 0.505 (0.083) | 0.511 (0.075) |
| V_PE.slp_ | - | - | - | - | 0.006 (0.001) | 0.007 (0.001) |
| r_PE_ | - | - | - | - | -0.614 (0.074) | -0.621 (0.061) |
|  |  |  |  |  |  |  |
| R | - | 0.485 (0.033)** | - | 0.619 (0.035)** | - | - |
| h^2^ | - | - | 0.170 (0.088)* | - | 0.142 (0.093) | 0.141 (0.075) |

**S6:** Effects of training on performance in the detour task – bivariate anlaysis

Fixed effects on each trait were as specified in the detour task and training trial univariate models in the main text. Random effects of *block* and *brood tank* were included and allowed to covary across traits while residual (observation level) covariance across traits is not statistically identifiable so was not estimated. Based on univariate modelling of training trial data we included random intercepts only for this trait (see results for justification), but we did include treatment specific variances for the detour task. This meant the among-individual variation was estimated as a 3x3 variance-covariance-correlation matrix (**ID**) in which the diagonal elements correspond to V_I_ in: (mean) performance in the training trials; (mean) performance in the detour task for treatment 1; and (mean) performance for treatment 2. Although all correlations in the matrix were estimated the data structure contains little information for the among-individual correlation (r_ID_) between treatment specific detour task performances (since each fish experienced only one treatment). While this parameter estimate will have high uncertainty, we do not constrain it to zero in the first instance since this may limit the model fit to unlikely parameter space (i.e., given 3 variables A, B, C, if, for instance r_AB_ and r_AC_ are strongly positive then it is unlikely that r_BC_ = 0). We then compared this to a simplified formulation in which the among-trait correlations in **ID** were all constrained to zero, providing a global test of among-individual correlation between traits. We then used an analogous multivariate animal model to estimate the genetic (**G**) and permanent environment (**PE**) components of the **ID** matrix.

**Table S6:** Character state representation of among-individual variation-correlation structure between trial specific performance in training trails (measured as *time to eat)*.

Table S4 above presents the estimated variance components and derived parameters for the six models of performance in the training trials. Based on the variance components from random regression Model B4, we use reaction norm (RN) intercepts and slopes. Assuming the assumption of linear reaction norms hold true this can be transformed to the corresponding ‘character state’ (CS) among-individual covariance matrix of trial specific performance time (designated **ID_CS_­**).

For a single trait (e.g., *time to eat*), **ID_CS_** =**Q·ID_RN_·Q^T^** (following e.g., equation 5.8 in Roff and Wilson 2015), where **ID_RN_** is the 2x2 covariance matrix of reaction norm (RN) intercepts and slopes, **Q^T^** is the transpose of matrix **Q**, and **Q** itself contains the values of the covariate (trial number) at which we wish to evaluate **ID_CS_**. Where we want **ID_CS_** to be a 9x9 matrix containing the among-individual variance in performance time at each trial number (T1-9) on the diagonal (shaded in grey), and the covariance between each pair of trial numbers in the off diagonal elements

**Q =** $\left[ \begin{matrix} 1 & 1 \\ 1 & 2 \\ 1 & 3 \\ 1 & 4 \\ 1 & 5 \\ 1 & 6 \\ 1 & 7 \\ 1 & 8 \\ 1 & 9 \end{matrix} \right]$

Following this, we transformed the estimated covariance matrix (**ID**) formulated under Model B4 above (i.e., with individual effects on performance in the training trials modelled as first order random regressions of trial number) to the corresponding character state matrix. This was then rescaled to yield point estimates of the among-individual correlation between trial specific performance. For simplicity we do not similarly attempt to transform estimates of uncertainty, but note that this table is a mathematical consequence (and transformation) of the statistical estimated presented in Table S3b above (i.e., this is same set of results presented a different way).

| Trial number | |  |  |  |  |  |  |  |  |
| --- | --- | --- | --- | --- | --- | --- | --- | --- | --- |
|  | T1 | T2 | T3 | T4 | T5 | T6 | T7 | T8 | T9 |
| T1 | 0.516 |  |  |  |  |  |  |  |  |
| T2 | 0.994 | 0.464 |  |  |  |  |  |  |  |
| T3 | 0.976 | 0.993 | 0.426 |  |  |  |  |  |  |
| T4 | 0.941 | 0.972 | 0.992 | 0.401 |  |  |  |  |  |
| T5 | 0.890 | 0.933 | 0.968 | 0.992 | 0.390 |  |  |  |  |
| T6 | 0.823 | 0.876 | 0.927 | 0.966 | 0.991 | 0.392 |  |  |  |
| T7 | 0.743 | 0.891 | 0.871 | 0.925 | 0.967 | 0.992 | 0.407 |  |  |
| T8 | 0.656 | 0.732 | 0.805 | 0.872 | 0.928 | 0.969 | 0.992 | 0.436 |  |
| T9 | 0.568 | 0.651 | 0.734 | 0.812 | 0.881 | 0.935 | 0.973 | 0.994 | 0.478 |

References

1. Becher SA, Russell ST, Magurran AE. Isolation and characterization of polymorphic microsatellites in the Trinidadian guppy (Poecilia reticulata). Mol Ecol Notes. 2002 Dec;2(4):456–8.

2. Bergero R, Gardner J, Bader B, Yong L, Charlesworth D. Exaggerated heterochiasmy in a fish with sex-linked male coloration polymorphisms. Proc Natl Acad Sci U S A [Internet]. 2019 [cited 2021 Jan 25];116(14):6924–31. Available from: https://doi.org/10.5061/dryad.70bs72s

3. Jones OR, Wang J. COLONY: A program for parentage and sibship inference from multilocus genotype data. Mol Ecol Resour [Internet]. 2010 May [cited 2021 May 7];10(3):551–5. Available from: www.cephb.fr/en/cephdb/

4. Wang J, Santure AW. Parentage and sibship inference from multilocus genotype data under polygamy. Genetics [Internet]. 2009 [cited 2021 Feb 4];181(4):1579–94. Available from: https://academic.oup.com/genetics/article-abstract/181/4/1579/6081211

5. Kalinowski ST, Taper ML, Marshall TC. Revising how the computer program CERVUS accommodates genotyping error increases success in paternity assignment. Mol Ecol [Internet]. 2007 Mar 1 [cited 2021 May 7];16(5):1099–106. Available from: https://onlinelibrary.wiley.com/doi/full/10.1111/j.1365-294X.2007.03089.x

6. Hammond RL, Bourke AFG, Bruford MW. Mating frequency and mating system of the polygynous ant, Leptothorax acervorum. Mol Ecol [Internet]. 2001 Nov 1 [cited 2021 May 7];10(11):2719–28. Available from: http://wbiomed.curtin.edu.au/genepop/

7. Hoffman JI, Amos W. Microsatellite genotyping errors: Detection approaches, common sources and consequences for paternal exclusion. Mol Ecol. 2005 Feb;14(2):599–612.
